# Immunogenicity of Calvenza-03 EIV/EHV^®^ Vaccine in Horses: Comparative In Vivo Study

**DOI:** 10.3390/vaccines9020166

**Published:** 2021-02-17

**Authors:** Selvaraj Pavulraj, Tobias Bergmann, Claudia Maria Trombetta, Serena Marchi, Emanuele Montomoli, Sidi Sefiane El Alami, Roberto Ragni-Alunni, Nikolaus Osterrieder, Walid Azab

**Affiliations:** 1 Institut für Virologie, Robert von Ostertag-Haus, Zentrum für Infektionsmedizin, Freie Universität Berlin, Robert-von-Ostertag-Str. 7-13, 14163 Berlin, Germany; pavulraj1vet@zedat.fu-berlin.de (S.P.); tobias-bergmann@gmx.de (T.B.); no.34@fu-berlin.de (N.O.); 2 Department of Molecular and Developmental Medicine, University of Siena, 53100 Siena, Italy; trombetta@unisi.it (C.M.T.); serena.marchi2@unisi.it (S.M.); montomoli@vismederi.com (E.M.); 3 VisMederi srl, 53100 Siena, Italy; 4 AL-REEF Stable, Abu Dhabi P.O. Box 5151, United Arab Emirates; sidisefiane@yahoo.com; 5 Equine Marketing Division, Boehringer Ingelheim META, Dubai P.O. Box 507066, United Arab Emirates; leprotto1961@yahoo.it; 6 Department of Infectious Diseases and Public Health, Jockey Club College of Veterinary Medicine and Life Sciences, City University of Hong Kong, Kowloon Tong, Hong Kong 999077, China

**Keywords:** horses, equine influenza, vaccination, immunogenicity, Florida clade 2

## Abstract

Equine influenza (EI) is a highly contagious acute respiratory disease of equines that is caused mainly by the H3N8 subtype of influenza A virus. Vaccinating horses against EI is the most effective strategy to prevent the infection. The current study aimed to compare the kinetics of EI-specific humoral- and cell-mediated immunity (CMI) in horses receiving either identical or mixed vaccinations. Two groups of horses were previously (six months prior) vaccinated with either Calvenza 03 EIV EHV^®^ (G1) or Fluvac Innovator^®^ (G2) vaccine. Subsequently, both groups received a booster single dose of Calvenza 03 EIV EHV^®^. Immune responses were assessed after 10 weeks using single radial hemolysis (SRH), virus neutralization (VN), and EliSpot assays. Our results revealed that Calvenza-03 EIV/EHV^®^-immunized horses had significantly higher protective EI-specific SRH antibodies and VN antibodies. Booster immunization with Calvenza-03 EIV/EHV^®^ vaccine significantly stimulated cell-mediated immune response as evidenced by significant increase in interferon-γ-secreting peripheral blood mononuclear cells. In conclusion, Calvenza-03 EIV/EHV^®^ vaccine can be safely and effectively used for booster immunization to elicit optimal long persisting humoral and CMI responses even if the horses were previously immunized with a heterogeneous vaccine.

## 1. Introduction

Equine influenza (EI) is a major cause of respiratory infection in horses worldwide. The infection in naïve horses is characterized by an incubation period of around two days followed by clinical signs of fever, depression, dry cough, and serous to mucopurulent nasal discharge, which cease after two weeks [1]. EI is extremely contagious and causes up to 100% morbidity; however, it is fatal in very young animals and immunocompromised old horses. Recently, continuous EI outbreaks have been reported in several countries, including USA, Europe, Asia, and African countries [2,3,4,5,6,7,8,9,10]. EI is caused by an orthomyxovirus of the genus influenza A virus that can undergo antigenic drift and shift to escape host immune defenses. The immunodominant viral glycoproteins hemagglutinin (HA) and neuraminidase (NA) play a major role in virus pathogenesis. Mutations that gradually accumulate over time in the HA epitope can alter the protein and reduce its binding to neutralizing antibodies, allowing more viruses to infect a large number of cells. Vaccination is the most effective strategy to protect against virus infection, alongside isolation, movement restriction, and basic biosecurity measures. Most equine organizations require vaccination of horses, at different intervals, to enter competitions and shows [11]. There are several whole-inactivated virus-based vaccines [12,13,14,15], subunit vaccines, canarypox vectored vaccines [16] and modified live viral vaccines that are commercially available (Table 1) [17,18,19,20]. Reverse genetic-based modified live-attenuated EI vaccines are currently under study [21,22]. Current vaccines are able to reduce clinical signs and viral shedding in vaccinated animals; however, sub-clinical shedding of the virus is a major problem [11]. In addition, disease outbreak associated with vaccine breakdown has been reported [23]. 

The World Organization for Animal Health (formerly Office International des Epizootics; OIE) expert surveillance panel on equine influenza virus (EIV) collects epidemiological data from clinical cases around the world to monitor the emergence of new strains and to have more information on the possible accumulation of mutations in HA epitopes to evaluate the current vaccines and to recommend new strain(s) inclusion whenever necessary [24]. All current EIV strains belong to the genus influenza A virus, subtype H3N8, that has evolved from the original Miami/63 and further evolved into the American and Eurasian lineage(s). The current circulating viruses evolved from the American/Florida sub-lineage and are clade 1 and 2 [25]. The OIE expert surveillance panel recommends incorporation of virus strains that represent both clade 1 (represented by A/equi-2/South Africa/03-like or A/equi-2/Ohio/03-like) and clade 2 (represented by A/equi-2/Richmond/07-like) sub-lineage viruses to confer optimal protection in immunized horses.

While challenge trials are generally regarded as the best method to ascertain vaccine efficacy, direct correlation has been established between vaccine efficacy and single radial hemolysis (SRH) assay [1]. The SRH zone size of >65 mm^2^ has been well correlated with clinical and complete protection [26,27,28]. Protection against EIV is primarily attributable to antibodies that prevent infection and reduce transmission through neutralizing the virus [29]. Therefore, SRH and virus neutralization (VN) assays are the standard methods of detecting EIV-specific antibody response along with hemagglutination inhibition (HI) assay. Cell-mediated immunity (CMI) is also important for elimination of EIV-infected cells and conferring cross-protection against other strains. As CMI is difficult to measure, interferon gamma (IFN-γ) synthesis assay has been used as a CMI marker in immunized and/or infected horses [30].

Most of the vaccine efficacy studies were performed on only a small number of animals, given the high costs involved in raising and keeping the horses. Studies representing the field conditions of a mixed (different) vaccination and data regarding EIV-specific immune status several weeks to several months after immunization are lacking. In the current study, we aimed to investigate the immune responses against EIV in two groups of horses previously vaccinated with either Calvenza 03 EIV EHV^®^ (G1) or Fluvac Innovator^®^ (G2). Horses, of both groups, were at the end of the duration of immunity (DOI) period, six months, required by the Fédération Équestre Internationale (FEI) when they received a booster single dose of Calvenza 03 EIV EHV^®^. Antibody titer was determined by both SRH and VN assays. On the other hand, CMI was determined by enzyme-linked immune absorbent spot (EliSpot) assay to measure the level of IFN-γ secretion. 

## 2. Materials and Methods 

### 2.1. Ethical Approval/Animal Welfare

This study was carried out with the consent of the farm-supervising veterinarian and did not alter the regular vaccination and blood sampling of horses. Immunization and sample collection were performed in the equine stable. Horses were in the off-racing season during the trial, housed in their stable, and provided with regular off-season rations and ad libitum water. Following immunization and sample collection, horses were carefully monitored for development of any adverse signs of illness. 

### 2.2. Vaccine

Commercially available Calvenza-03 EIV/EHV^®^ inactivated vaccine (Boehringer Ingelheim; Table 1) contains Florida sub-lineage clade 1 (A/Equi-2/Ohio/03), Kentucky (A/Equi-2/Kentucky/95), and Eurasian (A/Equi-2/Newmarket/93) strain of equine influenza and EHV-1 strain of equine respiratory isolate (souche KyA) was adjuvanted with Carbimmune and used to vaccinate a group of horses. Fluvac Innovator^®^ inactivated vaccine (Zoetis LLC, Parsippany, NJ, USA; Table 1) contains A/equi-2/ Kentucky/97, EHV-1, and EHV-4 and was adjuvanted with MetaStim^®^ and used to vaccinate another group of horses. 

### 2.3. Experimental Animals

**Figure 1:**
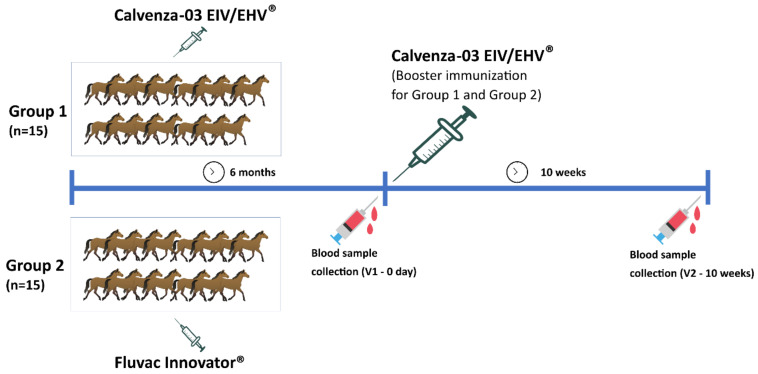
Vaccination and sampling regimen.

The study was carried out at the Al Reef Stable in the UAE. The stable at the time of the study had a population of around five hundred horses. Horses enrolled in the study were a mix of thirty endurance and flat racing purebred Arabian and Arabian crossing breeds, of various genders and from four to thirteen years of age, housed in the privately owned farm in the UAE. All horses in the off-racing season in the UAE were due for the semiannual influenza booster vaccination. The thirty horses were divided into two groups with fifteen horses in each (Figure 1). In group 1 (G1), horses were from 4 to 12 years of age and represented by 3 mares, 7 geldings, and 5 stallions. Horses in G2 were from 3 to 7 years of age and included 4 fillies and 11 colts. All animals were clinically healthy without any signs of illness. Each horse selected was previously vaccinated against EIV and equine herpesvirus (EHV) multiple times at six months intervals according to Emirates Equestrian Federation requirements and as per vaccine manufacturers’ recommendations, completing the basic vaccination with the same vaccine. It is worth noting that prior vaccination history of the horses before the study is not available.

### 2.4. Vaccination Protocol

G1: Horses were vaccinated with Calvenza-03 EIV/EHV^®^; 2 mL/intramuscular (im) on the side of the neck according to the manufacturer. After 6 months (day 0), the horses were boosted with Calvenza-03 EIV/EHV^®^; 2 mL/im. G2: Horses were vaccinated with Fluvac Innovator^®^; 1 mL/im on the side of the neck according to the manufacturer. After 6 months (day 0), the horses were boosted with Calvenza-03 EIV/EHV^®^, 2 mL/im.

### 2.5. Sampling and Shipping

Whole blood and serum samples were collected from all horses by jugular venipuncture two times: On Day 0 (V1—day 0) after 6 months from the last vaccination and before boosting with Calvenza-03 EIV/EHV^®^ and after 10 weeks (V2—10 weeks) of boosting (Figure 1). The samples were shipped in temperature-controlled (at 4 °C) shipments to Freie Universität Berlin, Department of Veterinary Medicine, Institute of Virology, Berlin, Germany, the OIE reference center for Equine Influenza.

### 2.6. Serology

Humoral immune response was evaluated by measuring serum antibody levels using SRH and VN assays against A/equi-2/Richmond/07.

#### 2.6.1. Single Radial Hemolysis

An SRH test was performed according to the protocol described by OIE, 2012 [31]. Briefly, the EIV antigen (A/equi-2/Richmond/07) was coupled with fixed sheep red blood cells (RBCs) suspended in agarose gel containing guinea pig complement. Wells were punched in the gel and filled with test and control serum. EIV specific antibodies and complement lysed the antigen-coated RBCs and resulted in a clear hemolytic zone, which is directly proportional to the titer of specific antibodies in the serum. Results were interpreted as zone of hemolysis in mm^2^ area [32]. As reported earlier, the SRH zone size of >65 mm^2^ was considered as the optimal level of antibody response for clinical and complete protection [26,27]. The assay was repeated two independent times.

#### 2.6.2. Virus Neutralization Assay

Madin–Darby Canine Kidney (MDCK) cells were cultured in Eagle’s Minimum Essential Medium (Sigma, St. Louis, MO, USA) supplemented with 2 mM L- Glutamine (Lonza, Milano, Italy), 1% non-essential amino acid solution, 100 U/mL penicillin-streptomycin mixture (Lonza, Milano, Italy), and 10% fetal bovine serum (Lonza, Milano, Italy), at 37 °C in a 5% CO_2_ humidified incubator. MDCK cells were seeded in 96-well plates using an UltraMDCK serum-free medium (Lonza, Milano, Italy), at a density of 2 × 10^4^ cells/well. A/equi-2/Richmond/07 live virus was titrated in serial 1-log dilutions to obtain a 50% tissue culture infective dose (TCID_50_). 

Serum samples were heat inactivated for 30 mins at 56 °C. Two-fold serial dilutions of serum samples were mixed with an equal volume of viral solution (100 TCID_50_/50 µL). After 1 h of incubation at 37 °C in a humidified atmosphere with 5% CO_2_, the serum–virus mixture was added to pre-seeded 96-well MDCK cell culture plates. The plates were incubated for 4 days at 37 °C in a humidified atmosphere with 5% CO_2_, after which the presence of cytopathic effect (CPE) was evaluated by means of an inverted optical microscope. A CPE more than 50% indicated infection. The VN titer was expressed as the inverse of the last serum dilution that showed inhibition of CPE.

### 2.7. Interferon Gamma Assay

The cellular immune response post vaccination was assessed by quantitatively measuring the frequency of IFN-γ-secreting peripheral blood mononuclear cells (PBMC) using ELISpot assay [33]. Briefly, 1 × 10^5^ PBMCs were incubated with 5 µg/mL concanavalin A (as positive control) or heat-inactivated A/equi-2/Richmond/07 EIV. In a 96-well plate with a polyvinylidene difluoride (PVDF) membrane, equine IFN-γ secreted from activated T cells was captured by immobilized, cross-reactive anti-bovine IFN-γ antibodies (Mabtech, Hamburg, Germany) and detected by a biotin-conjugated secondary anti-IFN-γ antibody (Mabtech, Hamburg, Germany) with a subsequent color reaction. Responses against the negative control (RPMI medium background counts) were subtracted from experimental values and statistical significance was assessed using the 2-way ANOVA test. All samples were tested in triplicates. A sample was considered positive if net spot-forming cells (SFCs) per one million cells exceeded 20, the stimulation index (SI) was greater than 2.0, and probability (*p* ≤ 0.05) was reached.

### 2.8. Statistical Data Analysis

The horses and the farm were unknown by the university personnel and the identity of the horses was masked by numbers generated by the attending farm veterinarian. Further, investigators were blinded to the vaccination status of the animals in both groups until the study and sample analyses were completed. Statistical analysis was carried out using GraphPad PRISM^®^ 5.01 software (San Diego, CA, USA). Normally distributed group samples were analyzed using 2-way repeated measures analysis of variance (ANOVA) with Tukey’s multiple comparisons post-test to test significant differences between groups. The Fisher’s exact test was used to compare the results of SRH and VN assays in each group. The level of significance was set at a *p*-value of ˂0.05.

## 3. Results

### 3.1. Clinical Signs

Horses immunized with both Calvenza-03 EIV/EHV^®^ and Fluvac Innovator^®^ vaccines did not develop either systemic or local adverse reaction following immunization. No signs of illness were recorded in any of the horses throughout the study. Only minor local reactions (swelling up to a few cm in diameter) to vaccination were observed and were resolved in subsequent days without intervention.

### 3.2. SRH Antibody Response to EIV

**Figure 2:**
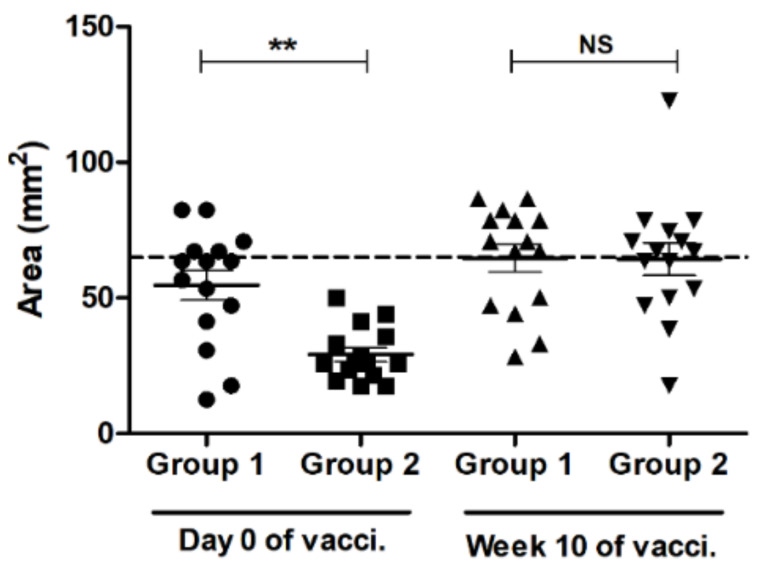
Mean EIV-specific antibody relative concentration determined by single radial hemolysis (SRH) assay. Serum samples were collected from groups 1 (G1) and 2 (G2) after six months of immunization (V1—day 0) with either Calvenza-03 EIV/EHV^®^ (Group 1; G1: black circles) or Fluvac Innovator^®^ (Group 2; G2: black squares) and after 10 weeks (V2—10 weeks) of booster immunization with Calvenza-03 EIV/EHV^®^ for horses of both groups (G1: upright black triangles; G2: downright black triangles). SRH assay was performed as described in the materials and methods. Total area of hemolysis was measured in mm^2^. The central line in the scattered dot plot indicates the mean of the data with standard error of mean (error bars). ** A significant increase (2-way repeated measures ANOVA with Tukey’s multiple comparisons post-test; *p* < 0.01) of hemolysis area for group 1 was seen when compared to group 2. NS—not significant; vacci.—vaccination.

SRH assay was performed to quantify the EIV-specific serum antibody level for both groups at the end of the DOI. In G1 (Calvenza-03 EIV/EHV^®^ immunized group) at V1 (day 0), five horses out of 15 still had protective SRH antibody in the serum (>65 mm^2^) [26], with an average of 54.69 mm^2^ for the whole group (see Figure 2 and Supplementary Materials). In contrast, none of the horses from G2 (Fluvac Innovator^®^ immunized group) had protective SRH antibody in the serum, with an average hemolysis zone of 29.13 mm^2^, which indicated that immunization with Calvenza-03 EIV/EHV^®^ maintained a protective antibody response in 33% for animals at six months post vaccination in comparison to the other vaccine.

In G1, booster immunization with Calvenza-03 EIV/EHV^®^ (at V2—10 weeks) stimulated higher antibody response, which persisted for a long duration, as reflected by a protective SRH level after 10 weeks of immunization with a mean hemolysis zone of 64.67 mm^2^ (Figure 2). In G2, although none of the horses had a protective SRH antibody level at the time of boosting (V1—day 0), booster vaccination with Calvenza-03 EIV/EHV^®^ vaccine resulted in a substantial increase in SRH antibody levels. The SRH antibodies persisted, as in the case of G1, to 10 weeks post immunization with a mean hemolysis zone of 64.29 mm^2^ (Figure 2), not substantially different from G1.

From these results, it can be concluded that the Calvenza-03 EIV/EHV^®^ vaccine can maintain protective antibody levels, in at least one third of the horses, for six months. Furthermore, when the horses have been immunized with another vaccine in the past, a single dose of Calvenza-03 EIV/EHV^®^ vaccine might be sufficient to ensure protective SRH antibody titer for at least 10 weeks post vaccination.

### 3.3. VN Assay Result

**Figure 3:**
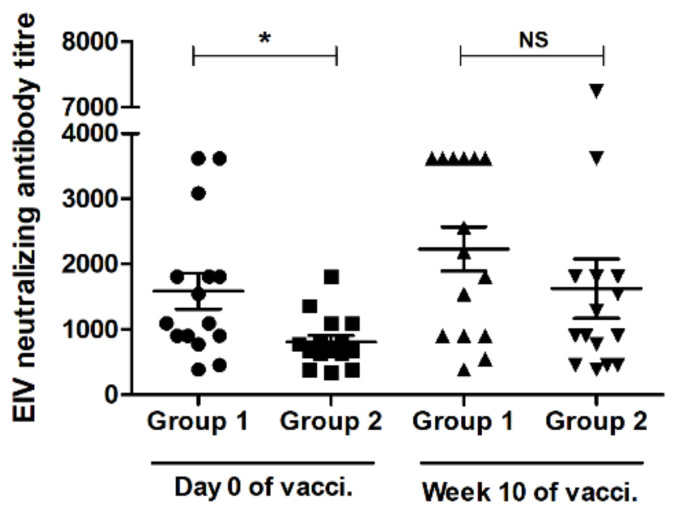
Mean EIV-specific serum neutralizing antibody titers determined by virus neutralization (VN) assay. Serum samples were collected from groups 1 (G1) and 2 (G2) after 6 months of immunization (V1—day 0) with either Calvenza-03 EIV/EHV^®^ (group 1; G1: black circles) or Fluvac Innovator^®^ (group 2; G2: black squares) and after 10 weeks (V2—10 weeks) of booster immunization with Calvenza-03 EIV/EHV^®^ for horses of both groups (G1: upright black triangles; G2: downright black triangles), and VN assay was performed. The central line in the scattered dot plot indicates the mean of the data with standard error of mean (error bars). * A significant increase (2-way repeated measures ANOVA with Tukey’s multiple comparisons post-test; *p* < 0.05) of antibody titer for group 1 was seen when compared to group 2. NS—not significant; vacci.—vaccination.

VN assay was performed to quantify the EIV-specific neutralizing antibody levels in the serum. Most of the animals in G1 (nine out of 15 animals) had a high neutralizing antibody level against EIV in serum (mean antibody titer of 1587.86) (Figure 3). A booster dose of Calvenza-03 EIV/EHV^®^ vaccine induced more virus neutralizing antibody responses and the levels were high even at 10 weeks post immunization with a mean antibody titer of 2231.13 (Figure 3). On the other hand, in G2, only four animals out of 15 had the EIV-neutralizing antibody (mean antibody titer of 806.73) before booster immunization, which increased to a mean titer of 1623.26 at 10 weeks post booster immunization. One animal in G2 had a very high level of neutralizing antibodies in serum; no clear interpretation.

Fisher’s exact test was performed to compare SRH and VN assay results for the serum samples tested from both G1 and G2 (Table 2). While minor variations were seen, no significant difference was observed between SRH and VN assay results.

### 3.4. Interferon Gamma Assay Result

**Figure 4:**
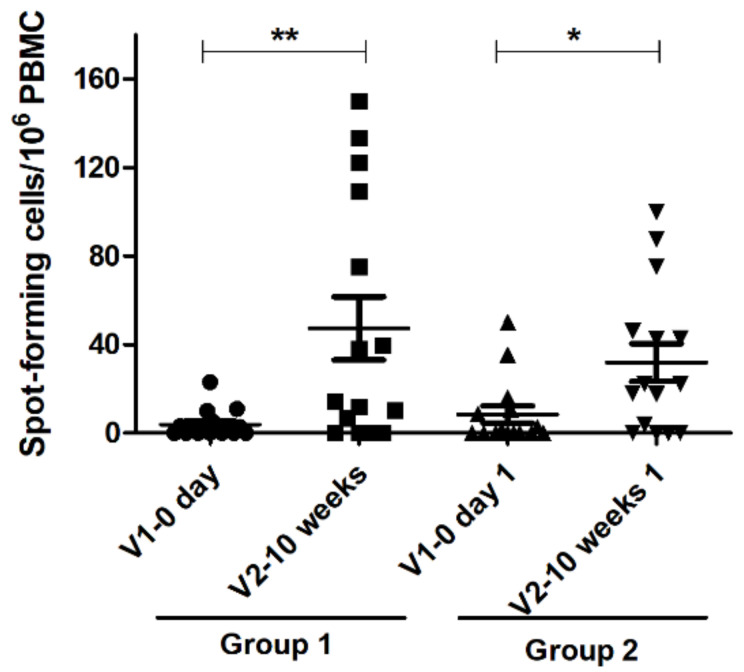
ELISpot analysis for interferon gamma (IFN-γ) secreting peripheral blood mononuclear cells (PBMC) from immunized horses. PBMC were collected from G1 and G2 horses before (day 0 = V1; G1: black circles; G2: upright black triangles) and after (10 weeks = V2; G1: black squares; G2: downright black triangle)) booster immunization. PBMC were isolated and stimulated with EIV. The response is presented as IFN-γ positive spot-forming cells per 10^6^ PBMC. The central line in the scattered dot plot indicates the mean of the data with standard error of mean (error bars). * *p* < 0.05; ** *p* < 0.01 using 2-way repeated measures ANOVA with Tukey’s multiple comparisons post-test.

IFN-γ assay was performed as an indirect indicator of stimulation of CMI response. After 6 months of the initial immunization, and prior to booster immunization, there were only very low to undetectable levels of IFN-γ-secreting PBMC in both G1 and G2 groups (Figure 4). The Calvenza-03 EIV/EHV^®^ vaccine at 10 weeks after booster immunization showed significant stimulation of CMI response as evidenced by the higher number of IFN-γ-producing PBMC. Comparing both groups, G1 revealed significantly higher (*p* ˂ 0.01) stimulation of CMI response. Taken together, it is evident that Calvenza-03 EIV/EHV^®^ immunization elicits higher CMI response for longer duration (10 weeks post booster immunization) despite primary immunization with a different EIV vaccine.

## 4. Discussion

Vaccination against EIV is essential to limit and prevent disease spread. While several vaccines are commercially available, the virus strain update as per the OIE expert surveillance panel for inclusion of both Florida clades 1 and 2 is lagging behind. These recommendations are aimed to prevent vaccine failure. Until EIV vaccines are updated to epidemiologically relevant virus strains, it is important to evaluate the protective efficacy of currently available vaccines against circulating field isolates. The whole-inactivated EIV vaccines were the first and still the most common type of vaccines used for several decades [34]. Evaluation of non-updated or partially updated vaccine performance in controlled studies and optimal conditions can determine if and/or how vaccines could be effective in the face of an outbreak. Kinetics of EIV vaccine responses are well documented [35]. A positive correlation between SRH antibody titers and the level of protection against EI has been demonstrated in several studies. While the vaccination schedule is similar between commercially available EIV vaccines, there were no recommendations for use of different EIV vaccines in the horse immunization schedule. However, the use of different commercial vaccines at different time points is a very common practice in the equine industry; this method is called the mixed regime [36]. Previous studies have already shown that the Calvenza-03 EIV/EHV^®^ vaccine induces the protective antibody titer immediately after immunization [29,36]. No clear data is available regarding the long-term persistence of EIV-specific humoral antibody response. The current study compared the kinetics of humoral immunity six months after initial immunization and 10 weeks after booster immunization. Based on SRH and VN antibody levels, horses immunized with Calvenza-03 EIV/EHV^®^ had significantly higher EIV-specific antibody than Fluvac Innovator^®^-immunized horses at six months post immunization. However, at 10 weeks post-booster immunization with Calvenza-03 EIV/EHV^®^, no difference between the two groups was observed. No clear correlation of protection has been established for the VN assay. VN titer at ≥64 was considered to correlate with protection against pyrexia caused by EIV [37]. About 10 weeks after Calvenza-03 EIV/EHV^®^ booster immunization, both G1 and G2 horses still had higher levels of VN titer of 2231 and 1623, respectively. Furthermore, Fisher’s exact test revealed a positive correlation between SRH and VN results among different groups without any significant difference. 

As mentioned earlier, assessment of IFN-γ-secreting PBMC has been used as a CMI marker in immunized horses [30]. Significant increase in EIV-induced IFN-γ-secreting PBMC was demonstrated in horses immunized with the canarypox-based EIV vaccine [30,38]. At 10 weeks after Calvenza-03 EIV/EHV^®^ booster immunization, IFN-γ-synthesizing PBMC was significantly increased in both G1 (*p* < 0.01) and G2 groups (*p* < 0.05). Protection against EIV has been previously observed in ponies with low or no SRH antibody levels at the time of infection, which supports the role of CMI in protection [39]. Stimulation of CMI in inactivated EIV vaccine immunized horses has been rarely investigated. The observed increase of CMI response in Calvenza-03 EIV/EHV^®^ immunized horses could also be correlated with the presence of carbimmune adjuvant in the vaccine [34]. We can see two main limitations of the current study: (i) No challenge with virulent EIV strain was possible due to the involvement of highly valuable endurance and flat racing purebred Arabian and Arabian cross breed horses; (ii) only horses from a single stud farm were included due to the high cost of raising horses in the UAE. While they were not within the scope of the present study, using naïve horses with various ages from different geographical locations, assessing immune response for longer duration after immunization (heterologous and homologous), and performing EIV and equine herpesvirus type 1 (EHV-1) challenge studies would be the potential areas of our future investigations.

## 5. Conclusions

In conclusion, between the two groups of horses previously vaccinated with different vaccines, the Calvenza-03 EIV/EHV^®^-immunized horses had significantly higher protective EIV-specific SRH and VN antibodies after six months of immunization. Furthermore, no significant difference was observed in antibody levels in vaccinated horses after booster immunization in both vaccine-identical G1 and non-identical G2. Booster immunization with the Calvenza-03 EIV/EHV^®^ vaccine significantly stimulated CMI response as evidenced by the significant increase in the frequency of IFN-γ-secreting PBMC. Therefore, the Calvenza-03 EIV/EHV^®^ vaccine can be safely and effectively used for booster immunization to elicit optimal long persisting protective humoral and CMI response even if the horses have previously been immunized with heterogeneous vaccines.

## Figures and Tables

**Table 1:** Currently available commercial equine influenza virus (EIV) vaccines.

S. No.:	Vaccine	Company	Adjuvant	Virus Strain	Type of Vaccine	Region in Use
1.	Calvenza-03 EIV/EHV^®^	Boehringer Ingelheim	Carbimmune	A/Equi-2/Newmarket/93 A/Equi-2/Kentucky/95 A/Equi-2/Ohio/03 EHV-1 souche KyA	Inactivated vaccine	USA
2.	Duvaxyn IE-T^®^	Elanco Animal Health	Carbomer, Aluminium hydroxide	A/Equi-1/Prague/56 A/Equi-2/Newmarket/93 A/Equi-2/Suffolk/89	Inactivated vaccine	Europe
3.	Equilis^®^ Prequenza-TE	MSD Animal Health	Saponin, Cholesterol, Phosphatidylcholine	A/Equi-2/South Africa/03 A/Equi-2/Newmarket/93	Inactivated vaccine	Europe
4.	Equi N Tect FLU^®^	Nisseiken Co Ltd.	Nil	A/equi-2/Ibaraki/1/07 A/equi-2/Yokohama/10	Inactivated vaccine	Japan
5.	Fluvac Innovator^®^	Zoetis LLC	MetaStim^®^	A/equi-2/ Kentucky/97 EHV-1 and EHV-4	Inactivated vaccine	USA
6.	Equip-FT^®^	Pfizer	ISCOM, Quillaic Acid derivative, Aluminium phosphate	A/Equi-1/Newmarket/77 A/Equi-2/Borlange/91 A/Equi-2/Kentucky/98	Subunit vaccine	Europe
7.	Flu Avert^®^ I.N.	MSD Animal Health	Nil	Cold adapted A/Equi-2/Kentucky/91	Modified live viral vaccine	Europe USA
8.	Proteqflu-TE^®^	Boehringer Ingelheim	Carbomer	A/Equi-2/Ohio/03 A/Equi-2/Richmond/07	Vector based canary pox vaccine	Europe

**Table 2:** Detection of EIV antibodies.

Group	Test	Positive	Negative	Total	Fisher’s Exact Test *p*-Value
G1: V1-0 day	SRH	5	10	15	0.2723; NS
	VN	9	6	15	
G1: V2 - 10 week	SRH	10	5	15	1.0000; NS
	VN	10	5	15	
G1: V1 - 0 day + V2 - 10 weeks	SRH	15	15	30	0.4348; NS
	VN	19	11	30	
G2: V1-0 day	SRH	0	15	15	0.0996; NS
	VN	4	11	15	
G2: V2 - 10 week	SRH	8	7	15	1.0000; NS
	VN	7	8	15	
G2: V1 - 0 day + V2 - 10 weeks	SRH	8	22	30	0.5796; NS
	VN	11	19	30	

Number of animals of groups 1 (G1) and 2 (G2) positive or negative for the presence of specific antibodies either by single radial hemolysis (SRH) or virus neutralization (VN) assays was detected. For SRH, zone of >65 mm^2^ and for VN, antibody titer of 1000 were considered as cutoff points. No significant difference in antibody levels was determined between the two assays (Fisher exact’s test). NS—not significant.

## Data Availability

The data presented in this study are available on request from the corresponding author. The data are not publicly available due to privacy regulations.

## Supplementary Materials

The following are available online at https://www.mdpi.com/2076-393X/9/2/166/s1, supplementary file.
